# Estrogens Can Disrupt Amphibian Mating Behavior

**DOI:** 10.1371/journal.pone.0032097

**Published:** 2012-02-15

**Authors:** Frauke Hoffmann, Werner Kloas

**Affiliations:** 1 Department of Ecophysiology and Aquaculture, Leibniz-Institute of Freshwater Ecology and Inland Fisheries, Berlin, Germany; 2 Department of Endocrinology, Institute of Biology, Humboldt-University Berlin, Berlin, Germany; Max-Delbrück Center for Molecular Medicine (MDC), Germany

## Abstract

The main component of classical contraceptives, 17α-ethinylestradiol (EE2), has high estrogenic activity even at environmentally relevant concentrations. Although estrogenic endocrine disrupting compounds are assumed to contribute to the worldwide decline of amphibian populations by adverse effects on sexual differentiation, evidence for EE2 affecting amphibian mating behaviour is lacking. In this study, we demonstrate that EE2 exposure at five different concentrations (0.296 ng/L, 2.96 ng/L, 29.64 ng/L, 2.96 µg/L and 296.4 µg/L) can disrupt the mating behavior of adult male *Xenopus laevis.* EE2 exposure at all concentrations lowered male sexual arousal, indicated by decreased proportions of advertisement calls and increased proportions of the call type rasping, which characterizes a sexually unaroused state of a male. Additionally, EE2 at all tested concentrations affected temporal and spectral parameters of the advertisement calls, respectively. The classical and highly sensitive biomarker vitellogenin, on the other hand, was only induced at concentrations equal or higher than 2.96 µg/L. If kept under control conditions after a 96 h EE2 exposure (2.96 µg/L), alterations of male advertisement calls vanish gradually within 6 weeks and result in a lower sexual attractiveness of EE2 exposed males toward females as demonstrated by female choice experiments. These findings indicate that exposure to environmentally relevant EE2 concentrations can directly disrupt male mate calling behavior of *X. laevis* and can indirectly affect the mating behavior of females. The results suggest the possibility that EE2 exposure could reduce the reproductive success of EE2 exposed animals and these effects might contribute to the global problem of amphibian decline.

## Introduction

The estrogen 17α-ethinylestradiol (EE2) is a main component of many classical contraceptives. In the EU around 50 kg of EE2 are produced each year and the prescription rate of this drug is very high [1]. In the U.S. it is assumed that 88 kg EE2 per year are used [2]. EE2 inhibits ovulation [3] by suppressing follicle stimulating hormone (FSH) secretion and altering structures of the endometrium [4], [5]. Unfortunately, EE2 is also a compound of high concern, because it is excreted unmetabolized through faces and urine [6], [7] and enters the environment via wastewater effluents [8]. It displays high estrogenic activity even at extremely low concentrations [9], [10] and has been detected in effluents [11]–[13] and surface waters [11], [14], [15] at concentrations ranging from 7–64 ng/L and 0.1–30 ng/L, respectively. Moreover, EE2 could even be detected in drinking water at concentrations of up to 1.4 ng/L [16], [17].

Consequently, aquatic vertebrates are main targets affected by the estrogenic action of EE2. In several fish species impairments of reproductive behaviors after short- and long-term exposure to EE2 were detected and assumed to result in selection against exposed fish [18]–[22]. Evidence for EE2 affecting amphibian mating behavior, however, is lacking, although amphibians are known as sentinels for environmental pollution especially concerning phenomena associated with endocrine disruption [23]: estrogenic and other endocrine disrupting compounds (EDCs) are assumed to contribute to the worldwide decline of amphibian populations e.g. via interferences with sexual differentiation [24], [25].

The aquatic anuran *Xenopus laevis* has been shown to be a suitable model organism for the screening of EDCs affecting reproductive biology *in vitro* [26], [27] and *in vivo* [25], [28], [29]. Because the preferred habitats of *X. laevis* are dark, turbid ponds, males rely on underwater acoustic cues to broadcast sexual arousal and location [30]. Each vocalization of *X. laevis* is composed of repetitive trills, consisting of trains of click sounds. Clicks are produced by contractions of laryngeal muscles [31] innervated by neurons of cranial nucleus IX-X within the vocal pathway, a defined neural circuit in the central nervous system (CNS), which is responsible for generating patterned vocal activity in the central vocal-motor pathway of *X. laevis* [32]. The vocalizations are highly stereotyped and thus different call types are distinguishable from each other with respect to spectral and temporal parameters [33], [34]: sexually aroused males predominantly produce advertisement calls (AC) and chirping, while sexually unaroused males predominantly produce growling, ticking and rasping calls [32], [35]. Female *X. laevis* have brief periods of sexual receptivity, during which they are attracted to and sexually stimulated by male AC [33].

Because male mate calling behavior of *X. laevis* is dependent on sex steroids, particularly androgens, as well as gonadotropins and prostaglandins [33], [36], [37], this behavior might be an appropriate endpoint for the assessment of estrogenic EDCs. Accordingly, we addressed three fundamental issues concerning the potential impact of EE2 on amphibian mating behavior using *X. laevis* as model organism. First, how exposure to the estrogenic EDC EE2 affects the calling behavior of male *X. laevis*; second whether modifications of male mating calls due to EE2 exposure are reversible over time; and third whether modifications of male mating calls by EE2 affect the attractiveness of these calls towards females by female choice.

## Methods

### EE2 exposure treatment

Adult frogs (five-year old) were kept in groups of up to 25 males within 60 L tanks at a water temperature of 20°C in the breeding stock of the Leibniz-Institute of Freshwater Ecology and Inland Fisheries (Berlin, Germany). They were fed a fish diet (Fisch-Fit; Interquell, Wehringen, Germany) twice a week and kept with a light∶dark cycle of 12∶12 h.

To test for effects of EE2 on male mate calling behavior of *X. laevis* we performed two test series, in which we exposed frogs to EE2 for 96 h at different environmentally relevant concentrations. In the first test series we determined the effects of EE2 at concentrations of 296 µg/L, 2.96 µg/L, and 29.6 ng/L (N = 10) and in the second test series we performed the EE2 treatments of 29.6 ng/L, 2.96 ng/L, and 0.296 ng/L (N = 10). A solvent (dimethyl sulfoxide, DMSO) control was used as vehicle control. For acclimation we transferred individual frogs into 60 L glass tanks (50×40×30 cm) containing 20 L of reconstituted tap water composed of distilled water supplemented with 5 g marine salt (Tropic Marin Meersalz, Tagis, Dreieich, Germany) three days before exposure. In the morning before exposure we injected males with 100 i.U. of human chorionic gonadotropin (hCG; dissolved in 50 µl distilled H_2_O) to stimulate a basic mate calling behavior [38]–[40]. We then dissolved EE2 (Sigma-Aldrich, Dreieich, Germany) in the solvent DMSO and exposed animals by adding the dissolved chemical to the ambient water [35]. After exposing stimulated males to EE2 we recorded the nocturnal calling behavior of the frogs for four consecutive nights as described previously [35]. During the experiment, frogs were fed a fish diet (Fisch-Fit; Interquell, Wehringen, Germany; 2.5 g/frog) twice a week and rearing water and chemicals were renewed every other day. Animals were anesthetized after the experiment using MS 222 (tricaine methanesulfonate) and weight and body length (snout to cloacae length) were measured. Afterwards, anesthetized frogs were sacrificed and liver samples were taken for Vtg mRNA expression measurements.

### Call analyses

Call analyses were performed as described previously [35] using Avisoft SasLab software (Avisoft, Berlin, Germany). Resulting data included the absolute calling activity of each male, as well as the relative proportions of the various call types uttered by each individual frog: advertisement calls (ACs) and chirping, a call type produced by clasping males; growling, a call type uttered by clasped males, ticking, which is produced by reproductively inactive males [33], [34] and rasping (Fig. S1), which is a call type previously shown to indicate a sexually unaroused state of the male [35]. Furthermore, we measured the following spectral and temporal parameters of the AC: mean duration of individual clicks, duration between individual clicks within one trill (ICI), mean click rate, mean number of clicks per call, duration of an entire call, mean peak and fundamental frequency, mean bandwidth, number of accentuated clicks, and the mean entropy of the calls [35]. Analyses were conducted blinded regarding treatment group. We used general linear mixed models (GLMMs) to analyze the overall treatment effect of EE2 on the different measured parameters of the male mate calling behavior. We set subjects as random factor and also included the covariates body weight, body length and water temperature in the model. We further analyzed parameters showing a significant variation between treatments using post-hoc pairwise comparisons (GLMMs) to determine where the variation existed. Raw data was not normally distributed; however, to reach the assumption of normally distributed residuals, normality of residuals was ensured using the Kolmogorov-Smirnoff test. To control for type I errors from conducting multiple tests, we applied false discovery rate (FDR) [41].

### Reversibility of alterations of AC

We tested reversibility of altered AC features by recording AC of male *X. laevis* (N = 10) stimulated by 100 IU hCG (dissolved in 50 µl distilled H_2_O) before and during a 96 h EE2 exposure (2.96 µg/L). After EE2 exposure frogs were kept without being exposed to any EDC. Four and six weeks after EE2 exposure we recorded and analyzed the nocturnal calling behavior of the frogs (restimulated with 100 IU hCG) again. Overall statistical differences were determined using Friedman tests. Differences between treatments were detected using two-tailed Wilcoxon signed-rank tests for paired samples. To control for type I errors from conducting multiple tests, we applied false discovery rate (FDR).

### Vitellogenin (Vtg) mRNA expression

We measured the expression of hepatic vitellogenin (Vtg) mRNA of the male frogs after the 96 h exposure period. Measuring Vtg induction is a classical method used as highly sensitive biomarker for the detection of estrogenic EDC [26], [42]. Vtg expression was determined from total RNA conducting real-time PCRs using a MX3000P real-time PCR cycler (Stratagene). We isolated total RNA using QIAzol Lysis Reagent (Qiazol Handbook, Qiagen). Prior to reverse transcription we treated RNA with DNase I (AmpGrade, Invitrogen). We then prepared cDNA using an oligo(dT) primer with 1 µg total RNA and analyzed 2 µl diluted cDNA (1∶5) by Real-Time PCR (SensiMix SYBR Low-Rox kit, Peqlab). Thermal cycler conditions and primers were applied as described previously [42]. We detected differences between treatment groups using One-way ANOVA followed by Dunnett T3 post-hoc tests. Normality of data was ensured using the Kolmogorov-Smirnoff test.

### Female choice playback experiments

We tested whether alterations of temporal or spectral parameters of male AC due to EE2 contamination might influence the attractiveness of exposed males towards females by performing female choice experiments in a y-maze apparatus (Fig. S2; water depth: 25 cm). We examined 30 female *X. laevis* (five-year old) stimulated with 600 IU hCG (dissolved in 100 µl dH_2_O). To determine whether receptive females can discriminate calls from noise and whether they prefer male calls over noise, females (N = 10) were given the choice between AC playback (N = 10) versus white noise in the first experiment. In the second female choice playback experiment females (N = 10) chose between two simultaneous AC playbacks. One of the two playbacks was derived from a male exposed to EE2 (N = 10) and the other playback was derived from the same male kept under control conditions. Here, all females were tested twice, using playbacks of a different male in the second trial of the same categories as before (EE2 exposed versus unexposed controls). In the third female choice experiment, females (N = 10) were presented with only one playback at a time: once an AC playback of a non-exposed control male (N = 10) and in another test trial a playback of AC uttered by the same male while being exposed to EE2 (N = 10). To control for potential confounding side preferences, playback order was counterbalanced between trials. All playbacks were derived from recordings of male AC produced in response to hCG stimulation. For ‘noise’ playbacks, we used white noise files without any vocalizations. A playback lasted 15 min and consisted of fifteen 1 min repetitions of a compilation of AC from a specific male. Every female received playbacks of a different male in each experiment/test trial to prevent pseudoreplication. Playbacks were standardized concerning the number of calls to avoid preferences on the basis of performance-related traits. On average ± SD playbacks had 847.6±35.3 advertisement calls with a frequency of 2111.2±114.0 kHz, bandwidth of 1748.3±434.5 kHz and an amplitude of −31.5±5.9 dB. None of these parameters differed significantly between the two treatments compared (Wilcoxon signed-ranks tests: P≥0.05). Playback tests took place in an insulated climate chamber in the dark. Experiments were digitally videotaped (OSCAR CCD Camera, 640×480 Pixel) with the help of an infrared headlight and analyzed in a ‘blind fashion’ regarding female identity and trial number.

To start a test trial we placed a female in a mesh cylinder at the single end of the y-maze (120 cm×100 cm and 20 cm high, Fig. S2). At the other end there were two speakers (UW30, frequency range 100–10,000 Hz; impedance 8 ohm), one in each arm of the y-maze (Fig. S2). Within the mesh cylinder females were able to hear playbacks from both arms. After an acclimation period of 90 s, playback(s) started. After another 30 s the cylinder was lifted from outside the chamber and females could move freely within the y-maze. For analyzing females' relative attraction we measured females' retention times in a 10 cm distance to a speaker and the time females moved when being close to a speaker (10 cm). We used nonparametric Wilcoxon signed-ranks tests for statistical analysis.

### Ethical note

The study was approved and permit granted by the German State Office of Health and Social Affairs (LaGeSo, Berlin, Germany; permit no. Reg. 0409/08). By habituating the animals to the experimental procedures, e.g. gentle handling beforehand, we minimized the stress to the animals during the experimental period.

## Results

### Mate calling behavior of male *X. laevis*

**Figure 1:**
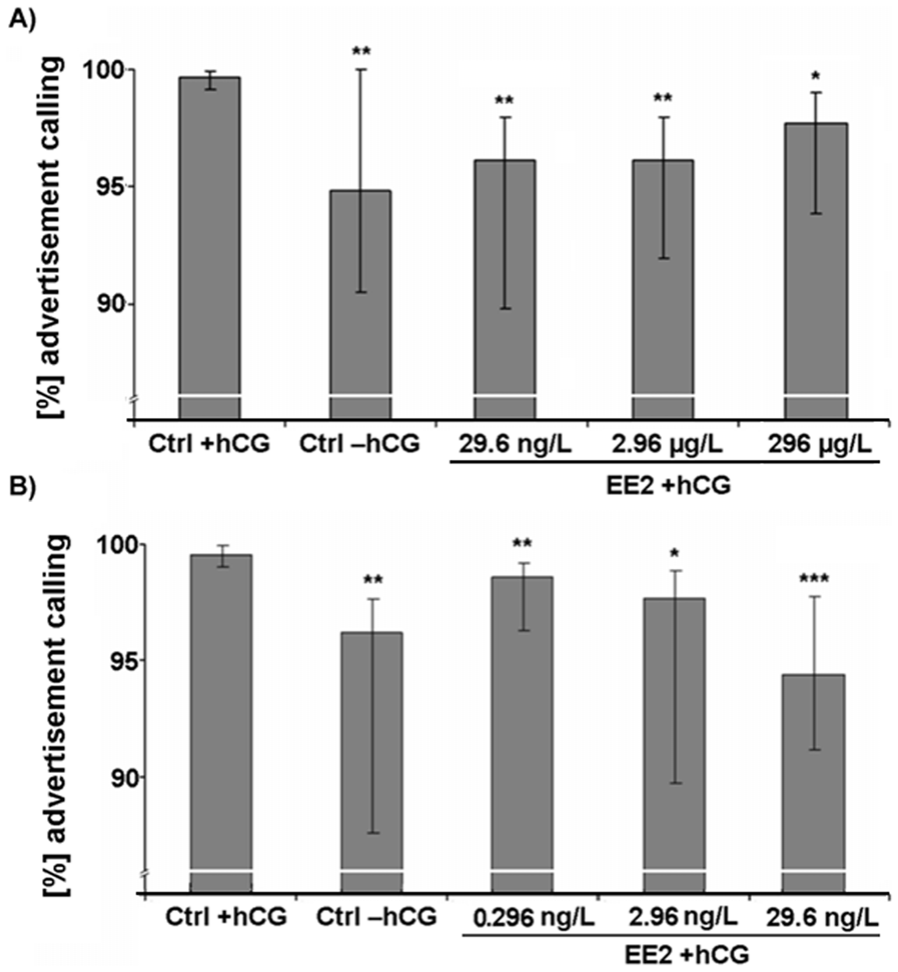
Percentages of advertisement calls. Median ± interquartile ranges (n = 10 per treatment) for EE2 exposure concentrations of A) 296 µg/L, 2.96 µg/L and 29.6 ng/L and B) 29.6 ng/L, 2.96 ng/L and 0.296 ng/L. Statistical differences were determined using General Linear Mixed models (GLMM). Significant differences from solvent control (CTRL) are marked by asterisks (* p≤0.05; ** p≤0.01; *** p≤0.001).

**Figure 2:**
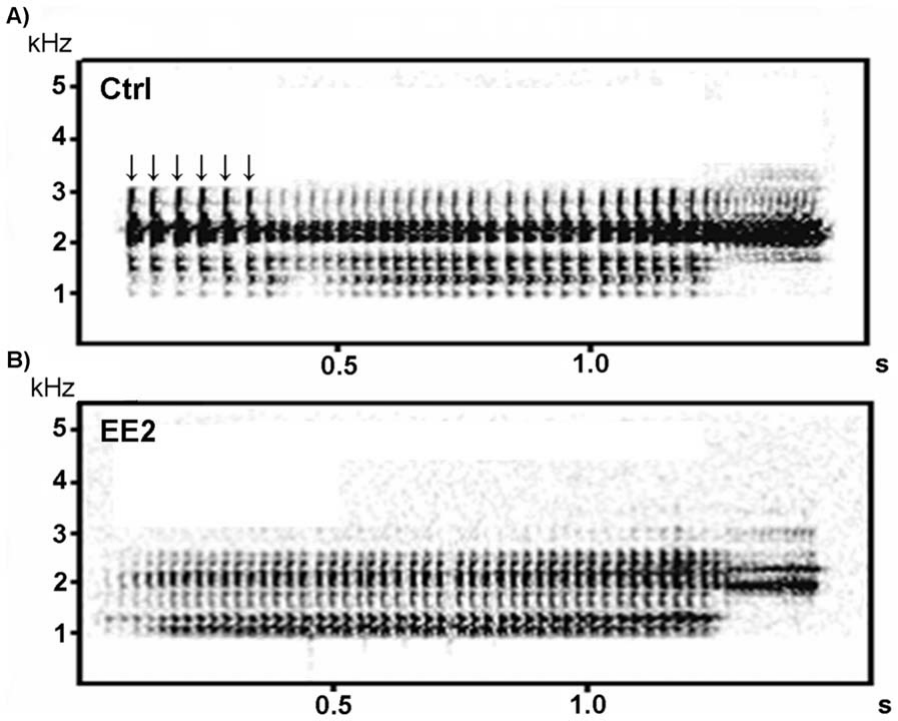
Spectrograms of advertisement calls. A) Advertisement call of an unexposed control male with six accentuated clicks at the beginning of the call, indicated by vertical arrows and B) advertisement call of an EE2 exposed male (2.96 µg/L) with no accentuated clicks at the beginning of the call.

**Figure 3:**
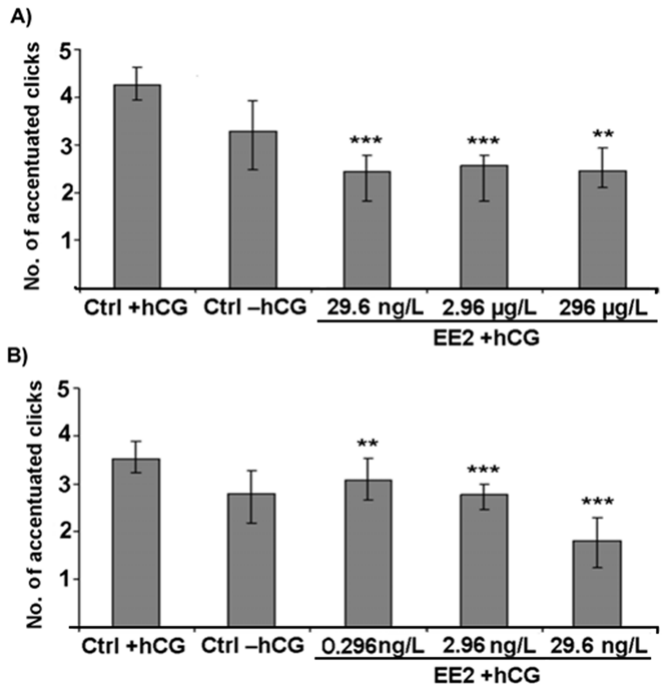
No. of accentuated clicks within male advertisement calls. Median ± interquartile ranges (n = 10 per treatment) for EE2 exposure concentrations of A) 296 µg/L, 2.96 µg/L and 29.6 ng/L and B) 29.6 ng/L, 2.96 ng/L and 0.296 ng/L. Statistical differences were determined using General Linear Mixed models (GLMM). Significant differences from solvent control (CTRL)+human chorionic gonadotropin (hCG) treatment are marked by asterisks (* p≤0.05; ** p≤0.01; *** p≤0.001).

**Figure 4:**
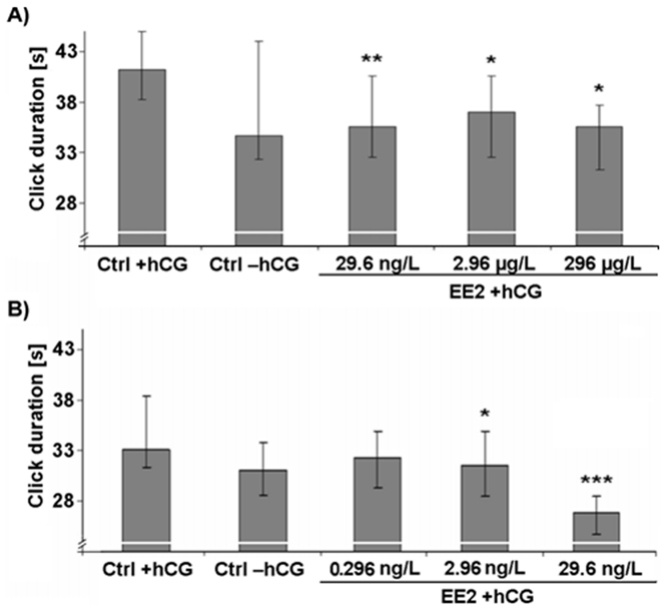
Duration of clicks of male advertisement calls. Median ± interquartile ranges (n = 10 per treatment) for EE2 exposure concentrations of A) 296 µg/L, 2.96 µg/L and 29.6 ng/L and B) 29.6 ng/L, 2.96 ng/L and 0.296 ng/L. Statistical differences were determined using General Linear Mixed models. Significant differences from solvent control (CTRL)+human chorionic gonadotropin (hCG) treatment are marked by asterisks (* p≤0.05; ** p≤0.01; *** p≤0.001).

Initially, we computed the absolute calling activity for each frog, but the total vocal output of the males was not affected by EE2 exposure (P>0.05; Table S1). Furthermore, we analyzed the composition of the produced vocalizations and determined the percentages of the different call types for each male. Interestingly, in comparison to unexposed frogs, EE2 exposed individuals generally uttered a significantly lower percentage of advertisement calls (P<0.05; Fig. 1 a and b) and a significantly higher proportion of rasping calls (P<0.05; Fig. S3 a and b). The other call types (chirping, growling and ticking) were not affected by EE2 exposure (Table S1). Additionally, we examined several characteristic temporal and spectral features of the AC. Analyses revealed that EE2 exposure at any of the five different concentrations resulted in significantly lower numbers of accentuated clicks at the beginning of the AC (P<0.01; Fig. 2 a and b, Fig. 3 a and b). The duration of clicks of AC was significantly reduced in all except the lowest treatment concentration (0.296 ng/L EE2) (P<0.05; Fig. 4 a and b). Body weight and body length, as well as water temperature did not affect any of these parameters.

### Reversibility of alterations of AC

**Figure 5:**
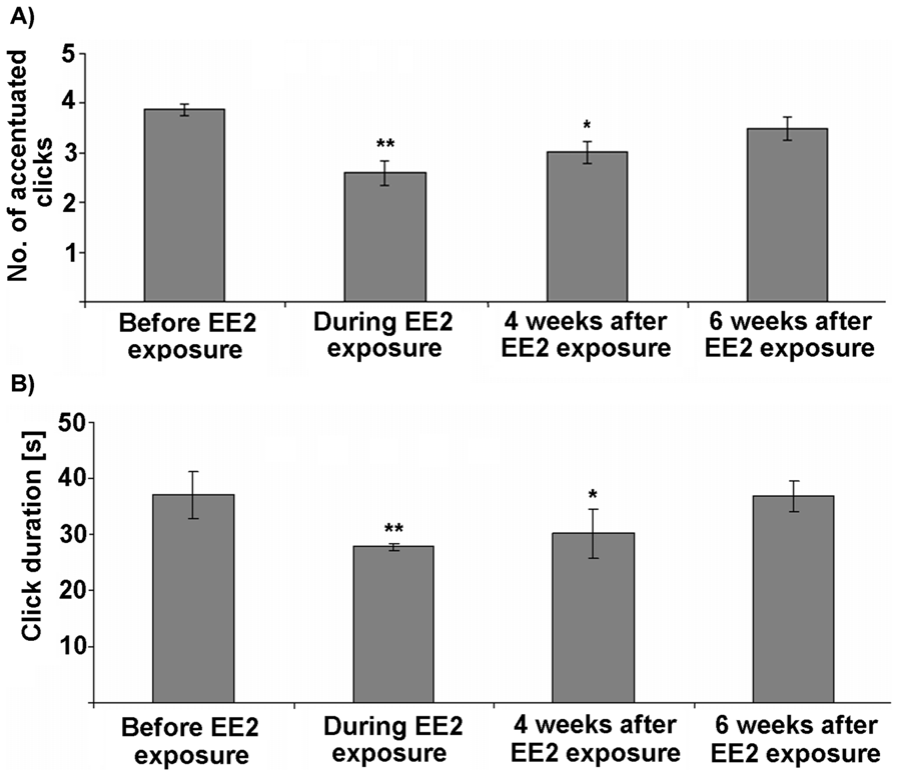
Reversibility of call modifications due to EE2 exposure. Median ± interquartile ranges (n = 10) of (A) no. of accentuated clicks and (B) duration of clicks within the advertisement calls before, during, and four and six weeks after EE2 exposure (at 2.96 µg/L). Statistical differences were determined using two-tailed Wilcoxon signed-rank tests for paired samples. To control for type I errors from conducting multiple tests, false discovery rate (FDR) was applied. Significant differences are marked by asterisks (* p≤0.05; ** p≤0.01; *** p≤0.001).

Having successfully shown that EE2 caused a reduced number of accentuated clicks and a decreased duration of clicks within male ACs, the question arises whether these alterations are reversible over time. Remarkably, these alterations due to EE2 exposure persisted during four weeks under control conditions without any EE2 (P<0.05; Fig. 5 a and b). After six weeks without EE2 exposure, however, the features did not show any differences to control levels any more (Fig. 5 a and b).

### Hepatic vitellogenin mRNA expression

EE2 contamination at concentrations of 296 µg/L and 2.96 µg/L significantly induced Vtg mRNA expression (P<0.01; Fig. S4). However, no lower EE2 concentration (29.6 ng/L) affected Vtg mRNA expression (Fig. S4).

### Biological relevancy of modified advertisement calls

When presented with recordings of male AC, female frogs were attracted to these vocalizations, as they spent more time near a speaker playing recorded male AC than a speaker playing only noise (median (IQR): 165.0 s (0 s–187.0 s) versus 0 s (0 s–0 s); P = 0.003). When having the choice between playbacks of AC of unexposed control males and playbacks of AC uttered by the same males while being exposed to EE2, female frogs were significantly more active when being close to the speaker playing AC of unexposed control males (first test trial: median (IQR): 16.0 s (1.5 s–22.0 s) versus 8.0 s (0 s–10.0 s), P = 0.049; second test trial: median (IQR): 5.5 s (0.5 s–10.5 s) versus 1.0 s (0 s–3.5 s), P = 0.027). Likewise, if presented with only one alternative playback at a time, females were also more active at the speakers when playbacks of control animals were played (median (IQR): 40.0 s (13.0 s–96.5 s) versus 20.0 s (5.5 s–40.5 s); P = 0.011). All subjects ovulated within 12 h after hCG injection, indicating a sexually aroused state.

## Discussion

In male *X. laevis* the relation between estrogen levels and courtship behavior is unclear. Estrogens were assumed to play no role in male calling activity [40], which was revised by these recent results. We found prompt and significant impacts of EE2 exposure on male mate calling behavior of *X. laevis* at environmentally relevant concentrations below the threshold of the classical and highly sensitive estrogenic biomarker Vtg induction. The reduction of AC proportions and the increase of proportions of the call type rasping indicate a lower sexual arousal of EE2 exposed males. This modification of behavior might be caused by altered relations between endogenous androgens and estrogens or disruptions of genomic or non-genomic signaling pathways [43].

Remarkably, EE2 exposure concentrations as low as 0.296 ng/L also alter spectral and temporal parameters of AC of male *X. laevis* and these immediate effects remain over four weeks. They are reversed to control levels only after six weeks, suggesting that these EE2 impacts might be due to fast and long term alterations in the central vocal-motor pathway located in the central nervous system, as it was shown for estrogen-exposed females [44], [45]. In birds, estrogens are assumed to contribute to the neural masculinization of the avian song system during a critical period of development [46], [47] and treatment of adult male starlings (*Sturnus vulgaris*) with estrogenic EDCs was shown to result in louder, longer and more complex birdsongs compared to control males [48]. This effect was suggested to be due to an increase in volume of the principle nucleus in the songbird brain, the HVC [48]. Accordingly, EE2 might similarly affect size and number of neurons within nuclei of the vocal pathway of male *X. laevis* [32] and thereby affect parameters of the male mate calling behavior, although the masculine pattern of muscle fibers necessary for song production in *X. laevis* are predominantly dependent on androgens [49]. Markman and colleagues [48] further demonstrated that female starlings prefer the more complex songs of males which were exposed to higher concentrations of estrogenic EDCs, although exposed males showed reduced immune function [48]. In contrast our findings demonstrate that female *X. laevis* are less attracted to AC of EE2 exposed males and perform courtship-specific behavior (positive phonotaxis) to a lesser extend when located in a pond with EE2 exposed males. Because sexual identity recognition of male and female frogs, as well as mate identification was shown to be based on temporal calling parameters [50]–[52], alterations of temporal or spectral parameters due to EE2 contamination might disable females to discriminate properly within and between *Xenopus* species. Moreover, spectral cues of calls of male *X. laevis* convey information about the attractiveness of a potential mate [52], thus modifications of spectral cues due to EE2 exposure might also be less attractive for selecting females.

According to our results, it was previously shown that courtship-specific behavior of male fish decreased after EE2 exposure at environmentally relevant concentrations [19]–[22] nearly as sensitive as for *X. laevis*. Saaristo and colleagues [19], [20], for instance, demonstrated that Sand goby (*Pomatoschistus minutus*) males exposed to EE2 show impaired courtship behavior (11 ng/L) [19] and fail in nest and mate competition (4 ng/L) [20]. Likewise, the frequency of courtship-specific behavior decreased in male zebrafish (*Danio rerio*) exposed to EE2 [21]. EE2 exposure (≥1 ng/L) even resulted in feminization of secondary sexual traits in male pipefish (*Syngnathus scovelli*), with males still being capable of reproduction, but females discriminating against exposed males [22]. These cumulative findings indicate that behaviors of aquatic vertebrates associated with courtship and mating can be adversely affected by extremely low concentrations of EE2, suggesting the possibility of a lower reproductive success of exposed animals. However, further studies are needed to clarify the impact of estrogenic EDC exposure on immune functions of male *X.* laevis. Moreover, further experiments are necessary to elucidate whether additional parameters of the male mate calling such as amplitude, bout lengths or temporal sequencing of different call types are also affected by estrogenic and other EDCs.

Similarly to EE2 exposed frogs we previously demonstrated [35] that AC of hCG stimulated male *X. laevis* exposed to the antiandrogen vinclozolin (VIN) had lower click durations and fewer accentuated clicks than AC of unexposed males. VIN and EE2 exposed animals also uttered lower proportions of advertisement calls, indicating a lower sexual aroused when compared with control frogs [35]. The only difference between EE2 and VIN treated animals was detected in the utilization of different call types: while EE2 treated frogs showed higher proportions of rasping, VIN treated animals uttered higher proportions of the call types growling and male ticking [35]. Hence, rasping seems to be affected by the amount of free circulating estrogens and/or the relation between androgen and estrogen levels, while growling and male ticking seem to be affected if androgen binding to the AR is competitively inhibited. Howsoever, this difference between treatments indicates that the newly established method can be used as a highly sensitive biomarker for the detection of EDCs, differentiating between different modes of action when detecting EDCs of antiandrogenic or estrogenic activity, which cannot be distinguished by determining sex ratio where estrogens and antiandrogens are changing sexual differentiation in same direction towards feminization.

In humans, EE2 at higher dosages (0.1 mg twice a day) has also been shown to reduce male sexual desire and activity [53], [54] and the decreasing male human reproductive health observed during the last few decades [54] was suggested to be, at least in part, attributable to exposure to estrogenic EDCs during fatal and childhood development [55], [56]. Nevertheless, further studies are needed to investigate, whether exposure to environmentally relevant concentrations of EE2 at different stages of development adversely affects sexual behavior of humans.

Taken together, our results indicate that exposure to EE2 at extremely low concentrations can directly disrupt male mate calling behavior of *X. laevis* and can adversely affect females' mating behavior in an indirect fashion, suggesting the possibility of a lower reproductive success which might contribute to the global problem of amphibian decline. Moreover, the high sensitivity and the capability of the method established here to differentiate between different modes of action when detecting EDC, as well as the potential to analyze vast datasets rapidly in a completely automated fashion indicate the huge potential for this rapid behavior test to become a sensitive, standardized, non-invasive biomarker with even diagnostic value.

## Supporting Information

Figure S1 Spectrogram of the call type rasping. This call type is defined as a long trill (>5 s), consisting of up to several hundreds of clicks in a frequency range between 1.8 and 2.3 kHz. The duration of a single rasping click ranges between 5 ms and 20 ms and the mean interclick interval within a rasping call lies between 15 ms and 100 ms.
(TIF)

Figure S2 Sketch of the Y-maze playback apparatus.
(TIF)

Figure S3 a and b: Percentages of the call type rasping, which indicates a sexually unaroused state of the male. Median ± interquartile ranges (n = 10 per treatment) for EE2 exposure concentrations of a) 296 µg/L, 2.96 µg/L and 29.6 ng/L and b) 29.6 ng/L, 2.96 ng/L and 0.296 ng/L. Statistical differences were determined using General Linear Mixed models. Significant differences from solvent control (CTRL)+human chorionic gonadotropin (hCG) treatment are marked by asterisks (* p≤0.05; ** p≤0.01; *** p≤0.001).
(TIF)

Figure S4 Vtg mRNA expression. Relative mRNA expression of hepatic vitellogenin in *X. laevis* after a five-day exposure to EE2 at three different concentrations (mean ± S.E.M.; n = 10 per treatment). Statistical differences were determined using One-way ANOVA followed by Dunnett T3 post-hoc tests. Normality of data was ensured using the Kolmogorov-Smirnoff test. Significant differences from control (CTRL)+human chorionic gonadotropin (hCG) are marked by asterisks (* p≤0.05; ** p≤0.01; *** p≤0.001).
(TIF)

Table S1 Effects of exposure to different concentrations of 17α-ethinylestradiol (EE2) on male calling behavior of *Xenopus laevis*. hCG injections were given in the morning before the first recording session. Values are median (interquartile range, IQR).
(DOC)
