# Supplementary figures and images for: Estrogens Can Disrupt Amphibian Mating Behavior
Source: PLoS One. 2012 Feb 15;7(2):e32097. doi: 10.1371/journal.pone.0032097 (PMC3280221; doi:10.1371/journal.pone.0032097)

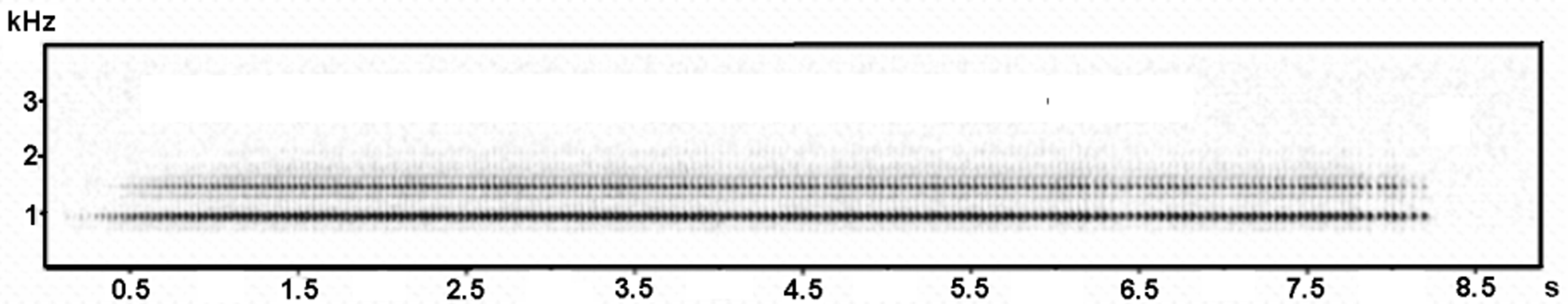

Supplement: Figure S1 — Spectrogram of the call type rasping. This call type is defined as a long trill (>5 s), consisting of up to several hundreds of clicks in a frequency range between 1.8 and 2.3 kHz. The duration of a single rasping click ranges between 5 ms and 20 ms and the mean interclick interval within a rasping call lies between 15 ms and 100 ms. (TIF) [file pone.0032097.s001.tif]

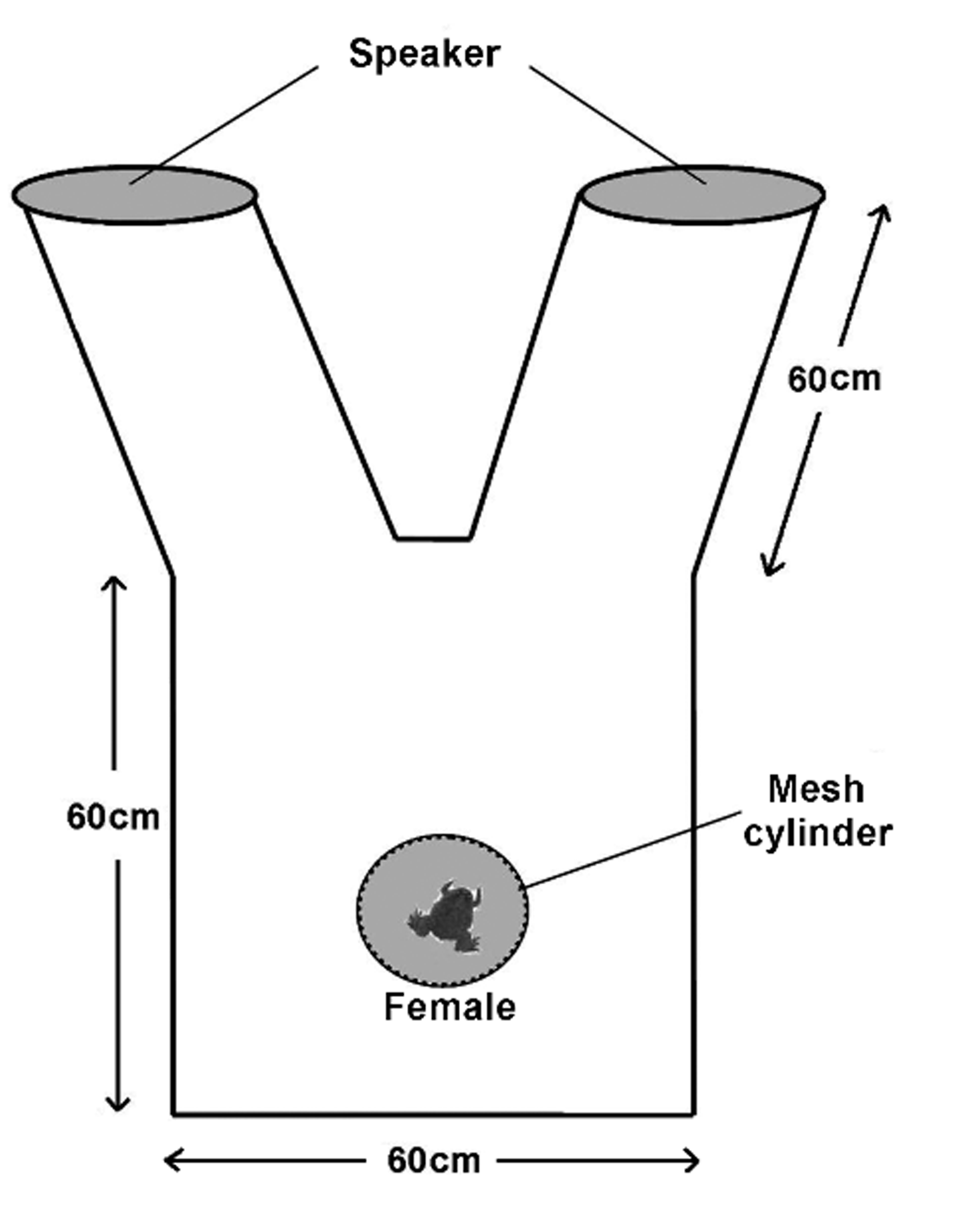

Supplement: Figure S2 — Sketch of the Y-maze playback apparatus. (TIF) [file pone.0032097.s002.tif]

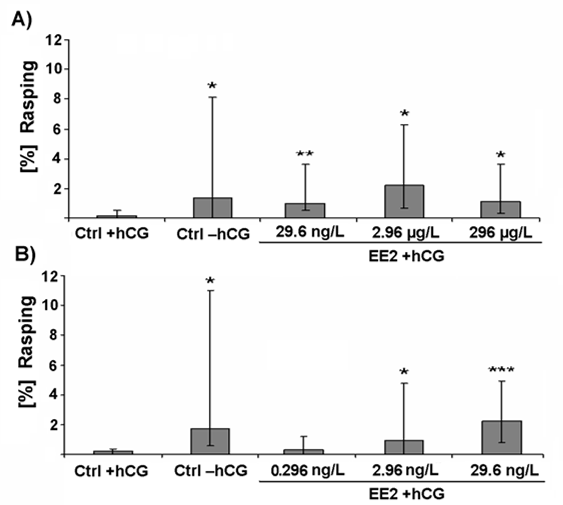

Supplement: Figure S3 — a and b: Percentages of the call type rasping, which indicates a sexually unaroused state of the male. Median ± interquartile ranges (n = 10 per treatment) for EE2 exposure concentrations of a) 296 µg/L, 2.96 µg/L and 29.6 ng/L and b) 29.6 ng/L, 2.96 ng/L and 0.296 ng/L. Statistical differences were determined using General Linear Mixed models. Significant differences from solvent control (CTRL)+human chorionic gonadotropin (hCG) treatment are marked by asterisks (* p≤0.05; ** p≤0.01; *** p≤0.001). (TIF) [file pone.0032097.s003.tif]

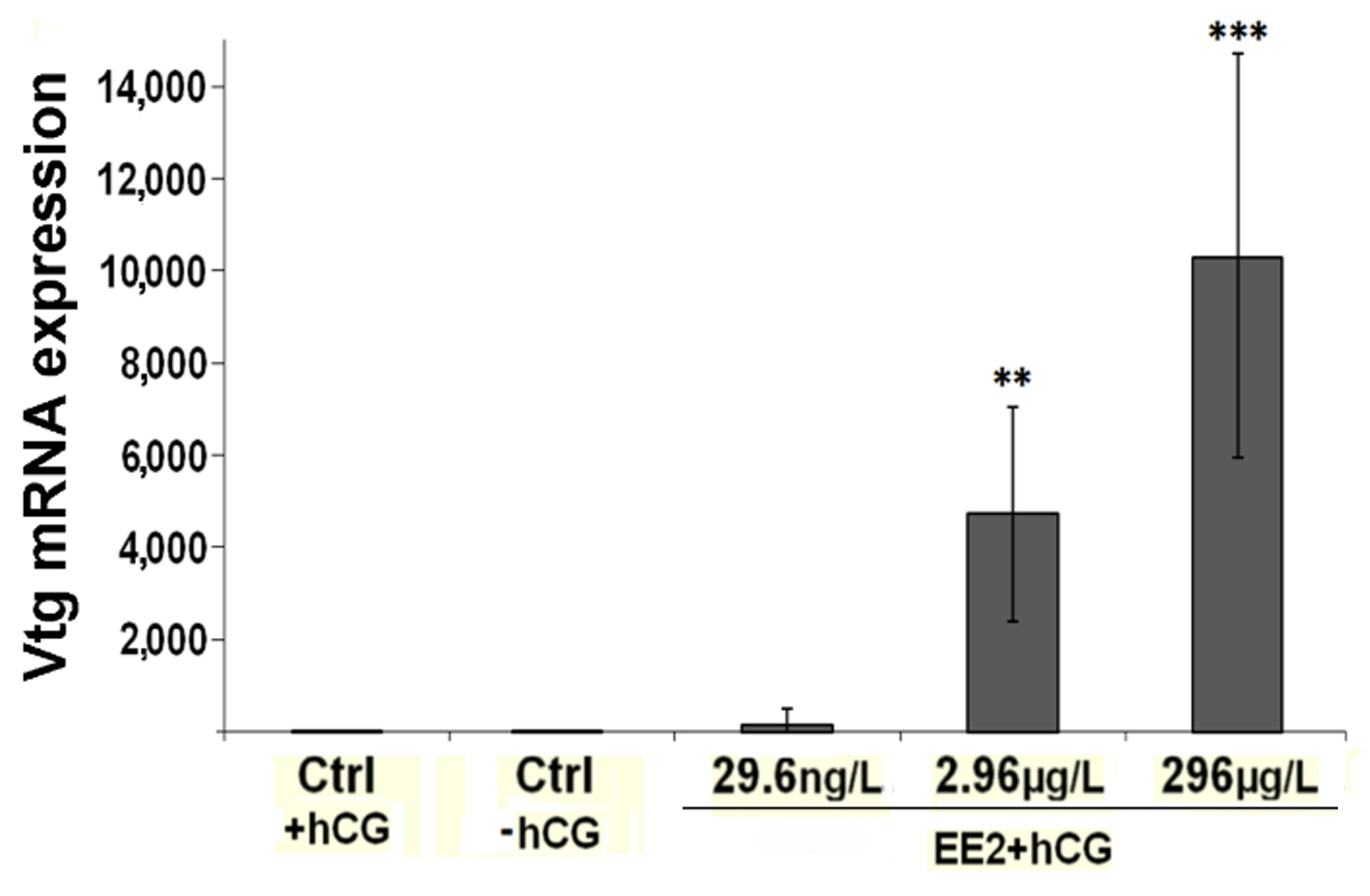

Supplement: Figure S4 — Vtg mRNA expression. Relative mRNA expression of hepatic vitellogenin in X. laevis after a five-day exposure to EE2 at three different concentrations (mean ± S.E.M.; n = 10 per treatment). Statistical differences were determined using One-way ANOVA followed by Dunnett T3 post-hoc tests. Normality of data was ensured using the Kolmogorov-Smirnoff test. Significant differences from control (CTRL)+human chorionic gonadotropin (hCG) are marked by asterisks (* p≤0.05; ** p≤0.01; *** p≤0.001). (TIF) [file pone.0032097.s004.tif]
