# Supplementary material for: Estrogens Can Disrupt Amphibian Mating Behavior
Source: PLoS One. 2012 Feb 15;7(2):e32097. doi: 10.1371/journal.pone.0032097 (PMC3280221; doi:10.1371/journal.pone.0032097)
Supplement: Table S1 — Effects of exposure to different concentrations of 17α-ethinylestradiol (EE2) on male calling behavior of Xenopus laevis. hCG injections were given in the morning before the first recording session. Values are median (interquartile range, IQR). (DOC) [file pone.0032097.s005.doc]

Tab. S1: Effects of exposure to different concentrations of 17α-ethinylestradiol (EE2) on male calling behavior of *Xenopus laevis*. hCG injections were given in the morning before the first recording session. Values are median (interquartile range, IQR).

| **Treatment** | | **Night**  (after exposure) | **Total vocal output**  (min) | **Chirping**  (%) | **Growling**  (%) | **Ticking**  (%) |
| --- | --- | --- | --- | --- | --- | --- |
| **First trial** | **Solvent control** | 1 | 456.0 (403.1 – 497.2) | 0.4 (0 – 0.1) | 0 (0 – 0.6) | 0 (0 – 0) |
|  |  | 2 | 365.8 (243.7 – 414.6) | 0.1 (0 – 0.4) | 0.1 (0 – 0.3) | 0 (0 – 0) |
|  |  | 3 | 331.8 (202.8 – 411.4) | 0 (0 – 0) | 0.1 (0 – 0.4) | 0 (0 – 0) |
|  |  | 4 | 258.1 (136.8 – 344.6) | 0.1 (0 – 0.6) | 0.2 (0 – 0.3) | 0 (0 – 0) |
|  | **EE2**  **29.64 ng/L** | 1 | 189.0 (70.5 – 357.5) | 0 (0 – 0) | 0.4 (0 – 0.7) | 0 (0 – 0) |
|  |  | 2 | 270.1 (124.4 – 402.6) | 0 (0 – 0) | 0.4 (0 – 2.0) | 0 (0 – 0) |
|  |  | 3 | 215.8 (79.1 – 339.0) | 0 (0 – 0) | 0.7 (0.1 – 1.7) | 0 (0 – 0) |
|  |  | 4 | 179.7 (90.4 – 220.8) | 0 (0 – 0) | 1.1 (0.1 – 2.8) | 0 (0 – 0) |
|  | **EE2**  **2.96 µg/L** | 1 | 318.9 (211.8 – 443.3) | 0 (0 – 0) | 0.4 (0 – 0.6) | 0 (0 – 0.5) |
|  |  | 2 | 159.7 (105.9 – 278.6) | 0 (0 – 0) | 0.004 (0 – 0.013) | 0 (0 – 0.8) |
|  |  | 3 | 102.8 (41.8 – 183.6) | 0 (0 – 0) | 1.0 (0.3 – 1.9) | 0.9 (0.4 – 1.3) |
|  |  | 4 | 91.7 (33.1 – 203.7) | 0 (0 – 0) | 1.3 (0.5 – 1.9) | 0.8 (0 –1.4) |
|  | **EE2**  **296.4 µg/L** | 1 | 240.3 (10.3 – 481.2) | 0 (0 – 0) | 0.2 (0 – 0.6) | 0.3 (0 – 1.6) |
|  |  | 2 | 208.2 (40.0 – 354.4) | 0 (0 – 0) | 0.2 (0 – 0.8) | 1.3 (0 – 2.2) |
|  |  | 3 | 160.0 (26.7 – 278.8) | 0 (0 – 0) | 0.5 (0.2 – 0.8) | 1.6 (0.2 – 2.7) |
|  |  | 4 | 37.7 (0.7 – 126.9) | 0 (0 – 0) | 0.1 (0 – 1.2) | 3.7 (0 – 7.3) |
| **Second trial** | **Solvent control** | 1 | 94.1 (6.0 – 179.3) | 0 (0 – 0) | 0 (0 – 0.1) | 0 (0 – 0) |
|  |  | 2 | 169.2 (41.4 – 225.1) | 0 (0 – 0.1) | 0.1 (0 – 0.3) | 0 (0 – 0) |
|  |  | 3 | 149.4 (74.5 – 237.5) | 0 (0 – 0) | 0 (0 – 0.6) | 0 (0 – 0) |
|  |  | 4 | 118.2 (39.5 – 176.7) | 0 (0 – 0) | 0.1 (0 – 0.2) | 0 (0 – 0) |
|  | **EE2**  **0.296 ng/L** | 1 | 129.4 (27.6 – 198.0) | 0 (0 – 0) | 0.5 (0.3 – 1.8) | 0 (0 – 0.2) |
|  |  | 2 | 136.1 (26.4 – 166.5) | 0 (0 – 0) | 0.4 (0.2 – 1.5) | 0 (0 – 0.1) |
|  |  | 3 | 139.5 (29.3 – 187.2) | 0 (0 – 0) | 1.1 (0.8 – 1.2) | 0 (0 – 0.1) |
|  |  | 4 | 50.9 (28.2 – 142.1) | 0 (0 – 0) | 0.9 (0.7 – 1.4) | 0.1 (0 – 0.2) |
|  | **EE2**  **2.96 ng/L** | 1 | 29.0 (3.4 – 231.5) | 0 (0 – 0) | 0.4 (0 – 1.7) | 0.1 (0 – 0.4) |
|  |  | 2 | 85.5 (8.9 – 265.1) | 0 (0 – 0) | 0.7 (0 – 1.4) | 0.1 (0 – 0.3) |
|  |  | 3 | 125.3 (63.3 – 263.0) | 0 (0 – 0) | 0.5 (0.2 – 1.2) | 0.1 (0 – 0.2) |
|  |  | 4 | 155.4 (32.0 – 235.0) | 0 (0 – 0) | 0.9 (0.2 – 4.5) | 0.1 (0 –0.1) |
|  | **EE2**  **29.64 ng/L** | 1 | 82.5 (39.2 – 135.5) | 0 (0 – 0) | 1.0 (0.8 – 3.0) | 0.2 (0.1 – 0.4) |
|  |  | 2 | 91.4 (47.5 – 166.5) | 0 (0 – 0) | 0.8 (0.5 – 1.5) | 0.4 (0 – 0.6) |
|  |  | 3 | 129.8 (95.7 – 181.6) | 0 (0 – 0) | 0.9 (0.5 – 2.1) | 0.3 (0.1 – 0.8) |
|  |  | 4 | 124.4 (50.1 –162.5) | 0 (0 – 0) | 1.2 (0.8 – 4.4) | 0.3 (0 – 0.7) |
